# Supplementary material for: Independent contributions of language activations in left and right temporal cortex to aphasia outcomes after stroke
Source: Imaging Neurosci (Camb). 2026 Apr 30;4:IMAG.a.1225. doi: 10.1162/IMAG.a.1225 (PMC13137568; doi:10.1162/IMAG.a.1225)
Supplement: Supplementary Material [file IMAG.a.1225_supp.pdf]

Independent contributions of language activations in left and right temporal cortex to aphasia outcomes after stroke

Sarah M. Schneck, Deborah F. Levy, Jillian L. Entrup, Melodie Yen, Dana K. Eriksson, Marianne Casilio, Anna V. Kasdan, Lily Walljasper, Caitlin F. Onuscheck, Larry T. Davis, Howard S. Kirshner, Michael de Riesthal, & Stephen M. Wilson

Supplementary material

Supplementary Table 1 Demographic and medical history data for all participants

PID	Group	Age	Sex	Hand	Educ	Stroke	TPO	Extent
0120	A	70-74	M	R	16	I	131.0	165.4
0131	A	70-74	M	R	18	I	116.8	134.3
0182	A	70-74	M	R	12	I	84.3	102.4
0190	A	55-59	M	R	13	I	63.9	144.2
0199	A	75-79	M	R	16	I	62.1	86.0
0308	A	50-54	M	R	16	I	43.1	97.7
0322	A	60-64	M	L	13	I	53.7	193.4
1155	A	65-69	F	R	13	H	103.6	11.4
1295	A	30-34	F	R	14	I	9.5	55.5
1346	A	70-74	M	R	20	I	11.8	20.9
1351	A	50-54	M	R	12	I	12.1	10.3
1354	A	65-69	M	R	16	H	62.5	17.5
1356	A	30-34	F	R	19	I	7.4	110.6
1361	A	65-69	F	R	15	I	82.4	53.3
1368	A	70-74	M	R	12	I	109.6	103.9
1535	A	50-54	M	R	12	I	14.0	191.3
1539	A	40-44	F	R	18	I	12.9	190.4
1554	A	45-49	F	R	15	I	14.2	67.3
1584	A	45-49	F	R	16	H	12.9	5.5
1594	A	40-44	M	R	14	I	12.7	29.9
1619	A	50-54	M	R	12	I	11.8	143.9
1649	A	50-54	M	R	12	I	3.3	31.9
1651	A	40-44	F	R	16	I	3.1	65.7
1656	A	80-84	F	R	12	I	11.9	16.3
1663	A	40-44	F	A	12	I	12.7	138.8
1671	A	40-44	M	R	12	H	12.3	17.1
1686	A	55-59	M	R	16	I	13.1	175.8
1702	A	80-84	M	R	12	I	13.1	85.3
1724	A	60-64	F	R	14	I	3.9	11.9
1731	A	45-49	M	R	18	I	33.1	237.2
1734	A	55-59	M	R	11	H	12.3	3.1
1738	A	70-74	M	R	14	I	101.7	164.9
1742	A	55-59	F	R	14	I	4.3	41.1
1776	A	60-64	M	R	12	I	13.1	3.8
1778	A	55-59	M	R	20	I	9.4	19.2
1812	A	75-79	M	R	12	I	3.2	35.4
1819	A	65-69	F	R	18	I	54.1	399.9
1826	A	75-79	M	R	12	I	2.8	2.1
1847	A	55-59	F	R	16	I	12.0	9.3
1859	A	75-79	F	R	9	I	4.6	11.1
1861	A	60-64	F	R	13	I	12.1	0.7
1894	A	60-64	M	L	10	I	3.2	13.9
1897	A	65-69	M	L	20	I	12.7	310.4

1915	A	50-54	F	L	15	I	2.9	4.9
1923	A	35-39	F	R	13	I	3.0	108.0
1934	A	75-79	M	R	12	I	3.4	7.0
1936	A	35-39	F	R	18	I	91.9	354.1
1940	A	55-59	F	L	12	I	12.5	25.8
1944	A	70-74	F	R	16	I	143.9	78.4
1970	A	75-79	F	L	6	I	2.9	89.9
1974	A	45-49	M	R	16	I	3.5	19.0
1977	A	50-54	M	R	10	I	3.0	15.9
1980	A	55-59	M	R	12	I	3.1	38.7
1981	A	65-69	F	R	14	I	3.7	179.6
1983	A	60-64	M	R	16	I	14.5	103.6
1996	A	40-44	M	R	19	I	15.6	3.0
2013	A	75-79	M	R	8	H	3.1	14.5
2014	A	30-34	M	R	12	H	3.4	18.8
2018	A	80-84	F	R	9	I	3.3	3.5
2026	A	45-49	M	L	12	I	12.4	26.5
2027	A	65-69	M	R	16	I	18.7	38.5
2039	A	55-59	M	R	16	I	12.1	139.1
2049	A	70-74	F	R	12	I	17.1	8.8
2066	A	60-64	M	R	16	I	17.5	17.5
2105	A	45-49	M	R	18	I	13.5	26.2
2201	A	30-34	F	R	12	I	14.0	336.6
2204	A	60-64	F	R	12	I	3.5	78.4
2220	A	60-64	M	R	11	I	5.0	174.5
2243	A	60-64	M	R	12	I	14.8	204.0
2364	A	70-74	M	A	20	I	259.4	434.6
1087	C	40-44	M	R	20	—	—	—
1200	C	70-74	F	R	16	—	—	—
1353	C	40-44	M	R	18	—	—	—
1362	C	35-39	M	R	14	—	—	—
1364	C	55-59	M	L	18	—	—	—
1369	C	25-29	M	L	16	—	—	—
1370	C	80-84	M	R	18	—	—	—
1371	C	35-39	M	R	18	—	—	—
1372	C	60-64	F	R	14	—	—	—
1373	C	55-59	F	R	16	—	—	—
1378	C	40-44	F	L	18	—	—	—
1381	C	50-54	F	R	18	—	—	—
1387	C	65-69	M	L	20	—	—	—
1388	C	60-64	M	R	20	—	—	—
1579	C	70-74	M	R	18	—	—	—
1580	C	55-59	F	R	14	—	—	—
1581	C	65-69	F	L	16	—	—	—
1582	C	75-79	F	R	14	—	—	—
1583	C	70-74	F	R	18	—	—	—

1587	C	75-79	F	L	20	—	—	—
1595	C	55-59	M	R	16	—	—	—
1607	C	35-39	M	R	12	—	—	—
1608	C	20-24	F	R	18	—	—	—
1621	C	45-49	M	R	18	—	—	—
1623	C	45-49	F	A	18	—	—	—
1624	C	60-64	F	R	16	—	—	—
1626	C	60-64	M	L	16	—	—	—
1627	C	55-59	F	R	16	—	—	—
1628	C	60-64	F	R	16	—	—	—
1740	C	60-64	M	R	20	—	—	—
1798	C	20-24	M	L	16	—	—	—
1811	C	50-54	F	R	18	—	—	—
1818	C	60-64	F	R	16	—	—	—
1827	C	60-64	F	R	14	—	—	—
1835	C	40-44	M	R	18	—	—	—
1839	C	40-44	M	R	16	—	—	—
1841	C	40-44	F	R	16	—	—	—
1842	C	40-44	F	R	16	—	—	—
1844	C	80-84	F	L	20	—	—	—
1849	C	60-64	F	R	16	—	—	—
1850	C	50-54	M	R	16	—	—	—
1851	C	40-44	F	R	16	—	—	—
1852	C	80-84	F	A	16	—	—	—
1873	C	20-24	F	R	17	—	—	—
1905	C	70-74	M	R	16	—	—	—

PID = Participant identifier; Group: A = Aphasia; C = Comparison; Sex: M = Male; F = Female; Hand = Handedness: R = Right; L = Left; A = Ambidextrous; Educ = Education (years); Stroke: I = Ischemic; H = Hemorrhagic; TPO = Time post onset (months); Extent = Lesion extent (cubic centimeters).

Supplementary Table 2 Quick Aphasia Battery subscores for all participants

PID	Group	WC	SC	WF	GC	SMP	Rep	Read	QAB	WWC	PPT
0120	A	9.86	3.47	4.17	6.96	8.33	6.53	5.14	6.24	9.58	14
0131	A	10	1.53	1.25	6.50	7.50	3.61	3.61	4.91	10	14
0182	A	9.58	5.28	2.50	0.38	0.83	4.44	3.20	4.02	10	14
0190	A	10	6.81	6.81	7.88	9.17	7.64	8.89	7.87	10	13
0199	A	10	7.09	4.58	3.46	0.00	4.17	4.45	5.31	10	14
0308	A	9.31	3.06	3.75	6.63	10	3.33	4.03	5.70	10	14
0322	A	5.42	0.83	0.17	0.04	0.00	3.34	0.00	1.42	5.83	13
1155	A	10	9.86	8.83	9.38	10	9.86	9.72	9.46	10	14
1295	A	9.86	7.78	7.94	5.42	8.33	6.25	6.11	7.41	10	14
1346	A	10	8.20	7.25	8.54	10	8.75	9.58	8.62	10	14
1351	A	8.89	2.22	6.64	7.96	10	4.72	6.80	6.49	10	14
1354	A	10	6.39	4.92	8.88	10	7.64	8.61	7.66	10	14
1356	A	9.30	1.39	2.42	0.79	0.00	2.78	1.80	3.15	10	14
1361	A	9.58	3.06	4.78	7.80	10	5.83	6.11	6.55	10	14
1368	A	9.44	6.81	7.78	8.55	10	7.64	9.86	8.23	10	14
1535	A	9.79	1.25	2.75	0.13	2.50	4.58	2.50	3.58	10	14
1539	A	9.17	7.92	6.50	7.38	5.00	7.92	7.92	7.36	8.75	14
1554	A	10	9.79	7.50	5.00	7.50	8.33	7.50	7.86	10	14
1584	A	10	10	7.50	9.63	10	9.17	9.58	9.29	10	14
1594	A	10	9.17	9.75	10	10	10	10	9.61	10	14
1619	A	8.96	0.21	3.00	5.00	2.50	4.17	0.83	3.94	7.08	14
1649	A	10	10	9.00	9.25	10	8.33	7.92	9.25	—	—
1651	A	10	10	7.00	9.25	10	9.17	9.58	8.91	—	—
1656	A	10	7.50	9.00	9.63	7.50	8.75	8.33	8.66	9.58	14
1663	A	9.79	10	8.75	9.75	10	9.17	7.08	9.24	10	14
1671	A	8.75	4.17	9.00	9.63	10	8.33	8.33	8.01	10	14
1686	A	8.33	2.50	0.00	0.00	0.00	2.50	0.00	2.15	5.83	14
1702	A	9.79	9.58	6.50	8.88	10	9.17	7.50	8.71	10	12
1724	A	10	10	9.50	9.38	7.50	8.75	9.17	9.27	10	14
1731	A	9.58	1.67	3.00	0.75	7.50	5.42	2.92	4.34	10	13
1734	A	9.38	7.71	7.75	9.63	10	7.50	7.50	8.40	7.50	14
1738	A	10	8.33	8.50	8.75	5.00	7.92	9.17	8.21	10	14
1742	A	9.17	6.04	8.50	9.50	10	8.75	9.58	8.56	10	14
1776	A	10	7.92	8.25	9.00	10	8.33	3.75	8.61	8.75	14
1778	A	10	10	7.75	8.25	10	9.17	8.33	8.88	10	14
1812	A	10	3.33	2.50	7.50	10	7.50	5.42	6.10	6.25	13
1819	A	4.58	0.00	0.00	0.63	7.50	5.00	0.42	2.37	1.67	11
1826	A	10	7.71	5.08	8.38	10	9.58	10	8.17	8.33	13
1847	A	9.79	8.75	8.75	9.63	10	9.17	9.58	9.10	10	14
1859	A	9.79	6.09	9.75	9.38	10	8.75	5.42	8.67	7.08	14
1861	A	10	10	9.33	9.50	10	9.17	9.17	9.49	10	14
1894	A	9.79	10	10	9.25	10	8.33	7.92	9.56	10	14
1897	A	4.38	0.63	0.00	0.00	0.00	0.00	0.00	0.90	0.00	11

1915	A	10	8.96	9.25	9.25	10	9.17	8.33	9.35	10	14
1923	A	8.33	3.33	2.75	6.50	10	5.00	0.83	5.33	9.58	14
1934	A	9.17	7.71	5.75	8.38	10	8.75	7.08	7.86	0.83	13
1936	A	9.79	4.38	5.25	4.63	10	4.17	5.83	6.21	7.92	13
1940	A	10	10	9.83	9.25	10	9.17	10	9.60	10	14
1944	A	10	8.54	7.83	8.25	7.50	9.17	9.17	8.24	10	14
1970	A	4.38	0.63	2.33	5.00	10	2.92	—	3.85	2.50	11
1974	A	9.79	9.58	10	9.25	10	8.33	9.00	9.57	10	14
1977	A	9.38	2.29	8.00	8.25	10	7.50	7.50	7.27	8.75	13
1980	A	10	4.17	4.42	7.00	10	5.00	7.50	6.48	8.33	14
1981	A	3.75	0.00	0.00	0.00	0.00	0.00	0.42	0.71	6.25	10
1983	A	10	8.96	8.08	8.50	7.50	8.33	8.75	8.43	10	14
1996	A	10	10	10	9.75	10	10	9.17	9.90	10	14
2013	A	9.79	9.17	8.08	9.50	10	9.17	9.17	9.13	10	14
2014	A	10	10	10	9.63	10	9.58	7.50	9.66	10	14
2018	A	10	9.17	9.75	9.38	10	8.75	9.17	9.56	9.58	14
2026	A	9.79	8.75	9.00	9.50	10	10	7.50	9.07	10	14
2027	A	10	10	10	9.75	10	10	9.17	9.90	9.58	14
2039	A	9.58	3.75	1.50	6.63	10	4.58	3.75	5.62	7.92	13
2049	A	10	9.79	9.00	9.75	10	9.17	10	9.51	10	14
2066	A	10	7.71	8.50	8.75	10	8.33	8.33	8.68	10	14
2105	A	10	9.79	10	9.75	10	10	9.17	9.86	10	14
2201	A	8.75	0.83	1.50	0.00	5.00	4.17	0.00	2.88	8.75	12
2204	A	7.92	4.79	7.00	7.13	10	5.00	7.92	6.78	10	14
2220	A	2.92	1.46	0.00	0.00	0.00	0.00	0.00	0.79	0.00	11
2243	A	6.88	1.04	1.00	0.00	0.00	2.50	0.00	1.77	2.92	11
2364	A	9.79	0.00	5.25	4.13	7.50	7.08	7.08	5.44	9.58	14
1087	C	10	10	10	10	10	10	10	10	—	—
1200	C	10	9.58	10	9.63	10	9.58	9.17	9.77	—	—
1353	C	10	10	10	10	10	10	10	10	—	—
1362	C	10	5.42	10	9.50	10	9.17	9.17	8.97	—	—
1364	C	10	10	9.00	10	10	10	10	9.86	—	—
1369	C	10	9.17	10	9.63	10	9.17	9.58	9.65	—	—
1370	C	10	9.58	10	10	10	10	10	9.92	—	—
1371	C	10	10	10	9.88	10	10	9.58	9.95	—	—
1372	C	10	9.17	9.25	10	10	10	10	9.74	—	—
1373	C	10	7.50	9.75	9.63	10	8.75	9.17	9.30	—	—
1378	C	10	10	8.50	9.50	10	10	10	9.72	—	—
1381	C	10	10	10	10	10	10	8.75	9.90	—	—
1387	C	10	10	10	9.75	10	9.17	10	9.90	—	—
1388	C	10	10	10	10	10	10	10	10	—	—
1579	C	10	10	10	10	10	10	10	10	—	—
1580	C	10	10	10	10	10	10	10	10	—	—
1581	C	10	10	10	10	10	10	10	10	—	—
1582	C	10	10	10	10	10	10	10	10	—	—
1583	C	10	10	10	10	10	10	10	10	—	—

1587	C	9.58	10	10	9.75	10	10	9.17	9.82	—	—
1595	C	8.75	10	10	9.75	10	9.17	10	9.67	—	—
1607	C	10	10	9.75	9.88	10	8.75	10	9.85	—	—
1608	C	10	10	10	10	10	10	10	10	—	—
1621	C	10	8.33	10	10	10	10	10	9.70	—	—
1623	C	10	9.58	10	10	10	10	10	9.92	—	—
1624	C	10	10	10	10	10	10	10	10	—	—
1626	C	10	6.67	9.75	9.88	10	9.58	10	9.31	—	—
1627	C	10	10	10	10	10	10	10	10	—	—
1628	C	10	10	10	10	10	10	10	10	—	—
1740	C	10	10	10	10	10	10	10	10	—	—
1798	C	10	10	10	10	10	10	10	9.79	—	—
1811	C	10	10	10	10	10	10	10	10	—	—
1818	C	10	8.75	9.00	10	10	10	10	9.64	—	—
1827	C	10	10	10	10	10	10	10	10	—	—
1835	C	10	10	10	10	10	10	10	10	—	—
1839	C	10	9.58	10	10	10	10	10	9.92	—	—
1841	C	10	10	10	10	10	10	10	10	—	—
1842	C	9.58	10	9.00	10	10	10	10	9.79	—	—
1844	C	9.58	8.75	10	10	10	10	10	9.70	—	—
1849	C	10	10	10	10	10	10	10	10	—	—
1850	C	10	9.17	10	10	10	10	10	9.85	—	—
1851	C	10	10	9.25	10	10	10	10	9.90	—	—
1852	C	10	8.75	10	10	10	10	10	9.78	—	—
1873	C	10	10	10	10	10	10	10	10	—	—
1905	C	10	9.58	10	10	10	10	10	9.92	—	—

PID = Participant identifier; Group: A = Aphasia; C = Comparison; WC = Word comprehension; SC = Sentence comprehension; WF = Word finding; GC = Grammatical construction; SMP = Speech motor programming; Rep = Repetition; Read = Reading aloud; QAB = Quick Aphasia Battery overall; WWC = Written word comprehension; PPT = Pyramids and Palm Trees—Pictures.

Supplementary Table 3 Between-groups comparison of left temporal activation

	Estimate	SE	*t*	*P*
(Intercept)	0.75711	0.13864	5.4609	3.0108e-07
Aphasia	-0.25102	0.040292	-6.23	8.9669e-09
Age	-0.0017091	0.0011818	-1.4462	0.151
SexF	-0.025318	0.034688	-0.72988	0.46703
HandLA	0.0071371	0.045326	0.15746	0.87517
Educ	-0.0091274	0.0065136	-1.4013	0.16397

Linear regression model: L_Temporal ~ 1 + Aphasia + Age + SexF + HandLA + Educ
Number of observations: 115, Error degrees of freedom: 109
Root Mean Squared Error: 0.182
R-squared: 0.306, Adjusted R-Squared: 0.274
F-statistic vs. constant model: 9.6, p-value = 1.3e-07

Supplementary Table 4 Between-groups comparison of left frontal activation

	Estimate	SE	*t*	*P*
(Intercept)	1.0928	0.18497	5.9083	4.004e-08
Aphasia	-0.21719	0.053755	-4.0405	9.963e-05
Age	-0.0030667	0.0015767	-1.9451	0.054342
SexF	0.066307	0.046279	1.4328	0.15478
HandLA	-0.068167	0.060471	-1.1273	0.26211
Educ	-0.019403	0.00869	-2.2328	0.027607

Linear regression model: L_Frontal ~ 1 + Aphasia + Age + SexF + HandLA + Educ
Number of observations: 115, Error degrees of freedom: 109
Root Mean Squared Error: 0.243
R-squared: 0.201, Adjusted R-Squared: 0.164
F-statistic vs. constant model: 5.47, p-value = 0.000157

Supplementary Table 5 Between-groups comparison of right temporal activation

	Estimate	SE	*t*	*P*
(Intercept)	0.15946	0.072479	2.2001	0.029909
Aphasia	-0.049196	0.021064	-2.3356	0.021344
Age	-0.0006178	0.00061781	-0.99998	0.31954
SexF	0.00042648	0.018134	0.023518	0.98128
HandLA	0.0027412	0.023696	0.11568	0.90812
Educ	0.002065	0.0034052	0.60644	0.54548

Linear regression model: R_Temporal ~ 1 + Aphasia + Age + SexF + HandLA + Educ
Number of observations: 115, Error degrees of freedom: 109
Root Mean Squared Error: 0.095
R-squared: 0.0981, Adjusted R-Squared: 0.0568
F-statistic vs. constant model: 2.37, p-value = 0.0438

Supplementary Table 6 Between-groups comparison of right frontal activation

	Estimate	SE	*t*	*P*
(Intercept)	0.1855	0.08394	2.2099	0.029199
Aphasia	-0.0080375	0.024395	-0.32948	0.74243
Age	-0.0013727	0.00071551	-1.9184	0.057675
SexF	0.065028	0.021002	3.0963	0.0024916
HandLA	0.045105	0.027443	1.6436	0.10314
Educ	-0.00014274	0.0039437	-0.036194	0.97119

Linear regression model: R_Frontal ~ 1 + Aphasia + Age + SexF + HandLA + Educ
Number of observations: 115, Error degrees of freedom: 109
Root Mean Squared Error: 0.11
R-squared: 0.141, Adjusted R-Squared: 0.102
F-statistic vs. constant model: 3.58, p-value = 0.00491

Supplementary Table 7 Initial correlation of left temporal activation with aphasia outcome

	Estimate	SE	*t*	*P*
(Intercept)	2.7403	2.1316	1.2855	0.20338
Age	0.015457	0.020707	0.74645	0.45822
SexF	0.30123	0.52295	0.57602	0.56669
HandLA	-0.159	0.7547	-0.21067	0.83383
Educ	0.099781	0.10058	0.99204	0.32504
StrHem	2.3536	0.86895	2.7085	0.0087241
LogTPO	-0.27629	0.24194	-1.142	0.25785
L_Temporal	8.7974	1.4048	6.2625	3.9881e-08

Linear regression model: QAB ~ 1 + Age + SexF + HandLA + Educ + StrHem + LogTPO + L_Temporal
Number of observations: 70, Error degrees of freedom: 62
Root Mean Squared Error: 2.08
R-squared: 0.443, Adjusted R-Squared: 0.38
F-statistic vs. constant model: 7.03, p-value = 3.6e-06

Supplementary Table 8 Initial correlation of left frontal activation with aphasia outcome

	Estimate	SE	*t*	*P*
(Intercept)	3.0758	2.4404	1.2604	0.21226
Age	0.020364	0.023382	0.87089	0.38717
SexF	-0.050523	0.60208	-0.083915	0.93339
HandLA	-0.057972	0.85242	-0.068009	0.946
Educ	0.097119	0.11361	0.85482	0.39594
StrHem	2.1425	0.97715	2.1926	0.032099
LogTPO	-0.30933	0.27222	-1.1363	0.26019
L_Frontal	4.5557	1.0774	4.2284	7.8838e-05

Linear regression model: QAB ~ 1 + Age + SexF + HandLA + Educ + StrHem + LogTPO + L_Frontal
Number of observations: 70, Error degrees of freedom: 62
Root Mean Squared Error: 2.34
R-squared: 0.294, Adjusted R-Squared: 0.214
F-statistic vs. constant model: 3.68, p-value = 0.00218

Supplementary Table 9 Initial correlation of right temporal activation with aphasia outcome

	Estimate	SE	*t*	*P*
(Intercept)	6.3893	2.5235	2.5319	0.013892
Age	0.011253	0.025534	0.44069	0.66097
SexF	0.24086	0.65558	0.3674	0.71457
HandLA	-0.49972	0.93041	-0.5371	0.59312
Educ	-0.0085561	0.12479	-0.068564	0.94556
StrHem	1.6035	1.0956	1.4636	0.14836
LogTPO	-0.35259	0.29796	-1.1833	0.24119
R_Temporal	7.9269	3.7177	2.1322	0.036956

Linear regression model: QAB ~ 1 + Age + SexF + HandLA + Educ + StrHem + LogTPO + R_Temporal
Number of observations: 70, Error degrees of freedom: 62
Root Mean Squared Error: 2.56
R-squared: 0.152, Adjusted R-Squared: 0.0564
F-statistic vs. constant model: 1.59, p-value = 0.155

Supplementary Table 10 Initial correlation of right frontal activation with aphasia outcome

	Estimate	SE	*t*	*P*
(Intercept)	6.4634	2.6643	2.4259	0.018192
Age	0.012489	0.027086	0.46107	0.64636
SexF	0.49067	0.72767	0.67431	0.50262
HandLA	-0.41988	0.98145	-0.42782	0.67027
Educ	0.032141	0.12778	0.25153	0.80224
StrHem	2.0985	1.1096	1.8912	0.063271
LogTPO	-0.3823	0.31134	-1.2279	0.22412
R_Frontal	0.16365	3.1403	0.052113	0.95861

Linear regression model: QAB ~ 1 + Age + SexF + HandLA + Educ + StrHem + LogTPO + R_Frontal
Number of observations: 70, Error degrees of freedom: 62
Root Mean Squared Error: 2.65
R-squared: 0.09, Adjusted R-Squared: -0.0127
F-statistic vs. constant model: 0.876, p-value = 0.53

Supplementary Table 11 Contribution of left temporal activation to aphasia outcome, above and beyond structural damage

	Estimate	SE	*t*	*P*
(Intercept)	-7.1316	2.094	-3.4058	0.0011824
Age	-0.015389	0.014667	-1.0492	0.29831
SexF	0.5928	0.36614	1.619	0.11069
HandLA	-0.27405	0.54504	-0.5028	0.61695
Educ	0.1194	0.069406	1.7204	0.09052
StrHem	0.69253	0.63643	1.0881	0.28088
LogTPO	2.0451	0.52541	3.8923	0.0002518
PredQAB	1.5589	0.22767	6.8471	4.5586e-09
LogTPOxPredQAB	-0.27089	0.069664	-3.8885	0.00025498
L_Temporal	4.8671	1.1087	4.39	4.6713e-05

Linear regression model: QAB ~ 1 + Age + SexF + HandLA + Educ + StrHem + LogTPO + PredQAB + LogTPOxPredQAB + L_Temporal
Number of observations: 70, Error degrees of freedom: 60
Root Mean Squared Error: 1.42
R-squared: 0.746, Adjusted R-Squared: 0.708
F-statistic vs. constant model: 19.6, p-value = 8.44e-15

Supplementary Table 12 Contribution of left frontal activation to aphasia outcome, above and beyond structural damage

	Estimate	SE	*t*	*P*
(Intercept)	-7.3492	2.3033	-3.1907	0.0022582
Age	-0.016852	0.016124	-1.0451	0.30016
SexF	0.44514	0.40934	1.0874	0.28119
HandLA	-0.16017	0.59579	-0.26883	0.78898
Educ	0.12096	0.076071	1.5901	0.11706
StrHem	0.348	0.68682	0.50668	0.61424
LogTPO	2.0459	0.57428	3.5625	0.00072698
PredQAB	1.6539	0.24711	6.6929	8.3365e-09
LogTPOxPredQAB	-0.26585	0.076134	-3.4918	0.00090665
L_Frontal	1.9893	0.78869	2.5222	0.014332

Linear regression model: QAB ~ 1 + Age + SexF + HandLA + Educ + StrHem + LogTPO + PredQAB + LogTPOxPredQAB + L_Frontal
Number of observations: 70, Error degrees of freedom: 60
Root Mean Squared Error: 1.56
R-squared: 0.697, Adjusted R-Squared: 0.651
F-statistic vs. constant model: 15.3, p-value = 1.39e-12

Supplementary Table 13 Contribution of right temporal activation to aphasia outcome, above and beyond structural damage

	Estimate	SE	*t*	*P*
(Intercept)	-6.6512	2.2653	-2.9361	0.0047063
Age	-0.023791	0.015762	-1.5094	0.13643
SexF	0.47181	0.40264	1.1718	0.24591
HandLA	-0.315	0.5916	-0.53245	0.59638
Educ	0.068972	0.076143	0.90583	0.36865
StrHem	-0.2336	0.68528	-0.34089	0.73438
LogTPO	2.0877	0.5697	3.6645	0.00052652
PredQAB	1.7412	0.24247	7.1808	1.2292e-09
LogTPOxPredQAB	-0.26603	0.075469	-3.525	0.00081768
R_Temporal	6.1781	2.2519	2.7435	0.008004

Linear regression model: QAB ~ 1 + Age + SexF + HandLA + Educ + StrHem + LogTPO + PredQAB + LogTPOxPredQAB + R_Temporal
Number of observations: 70, Error degrees of freedom: 60
Root Mean Squared Error: 1.54
R-squared: 0.702, Adjusted R-Squared: 0.657
F-statistic vs. constant model: 15.7, p-value = 8.45e-13

Supplementary Table 14 Contribution of right frontal activation to aphasia outcome, above and beyond structural damage

	Estimate	SE	*t*	*P*
(Intercept)	-7.1112	2.4516	-2.9007	0.005198
Age	-0.020749	0.016929	-1.2257	0.22511
SexF	0.51746	0.45083	1.1478	0.25561
HandLA	-0.3301	0.63731	-0.51796	0.60639
Educ	0.10419	0.07916	1.3162	0.1931
StrHem	0.078195	0.71116	0.10995	0.91281
LogTPO	2.0695	0.6021	3.4371	0.0010739
PredQAB	1.7733	0.25792	6.8754	4.0783e-09
LogTPOxPredQAB	-0.26751	0.080173	-3.3367	0.0014593
R_Frontal	1.7176	1.9437	0.88367	0.3804

Linear regression model: QAB ~ 1 + Age + SexF + HandLA + Educ + StrHem + LogTPO + PredQAB + LogTPOxPredQAB + R_Frontal
Number of observations: 70, Error degrees of freedom: 60
Root Mean Squared Error: 1.63
R-squared: 0.669, Adjusted R-Squared: 0.619
F-statistic vs. constant model: 13.5, p-value = 1.69e-11

Supplementary Table 15 Contribution of multivariable functional activation to aphasia outcome, above and beyond structural damage

	Estimate	SE	*t*	*P*
(Intercept)	-7.3309	2.0508	-3.5746	0.00071468
Age	-0.014668	0.014384	-1.0198	0.31208
SexF	0.37823	0.37149	1.0181	0.31284
HandLA	-0.31723	0.53195	-0.59635	0.55326
Educ	0.097719	0.068801	1.4203	0.16087
StrHem	0.41017	0.63612	0.64481	0.52159
LogTPO	2.0943	0.51174	4.0925	0.00013359
PredQAB	1.5641	0.22215	7.041	2.5017e-09
LogTPOxPredQAB	-0.27667	0.067843	-4.0781	0.00014017
L_Temporal	3.7535	1.2526	2.9966	0.0040124
R_Temporal	4.4921	2.0833	2.1562	0.035224
L_Frontal	0.80067	0.79449	1.0078	0.31775

Linear regression model: QAB ~ 1 + Age + SexF + HandLA + Educ + StrHem + LogTPO + PredQAB + LogTPOxPredQAB + L_Temporal + R_Temporal + L_Frontal
Number of observations: 70, Error degrees of freedom: 58
Root Mean Squared Error: 1.39
R-squared: 0.768, Adjusted R-Squared: 0.723
F-statistic vs. constant model: 17.4, p-value = 1.46e-14

Supplementary Table 16 Model predicting aphasia outcomes from structural damage

	Estimate	SE	*t*	*P*
(Intercept)	-6.6197	2.3834	-2.7775	0.0072683
Age	-0.023625	0.016583	-1.4246	0.15936
SexF	0.66736	0.41695	1.6006	0.11463
HandLA	-0.20828	0.6211	-0.33533	0.73852
Educ	0.10347	0.079013	1.3095	0.19528
StrHem	0.10447	0.70926	0.1473	0.88338
LogTPO	2.0252	0.59893	3.3813	0.0012636
PredQAB	1.7426	0.25511	6.8308	4.5219e-09
LogTPOxPredQAB	-0.25829	0.079348	-3.2552	0.0018512

Linear regression model: QAB ~ 1 + Age + SexF + HandLA + Educ + StrHem + LogTPO + PredQAB + LogTPOxPredQAB
Number of observations: 70, Error degrees of freedom: 61
Root Mean Squared Error: 1.62
R-squared: 0.665, Adjusted R-Squared: 0.621
F-statistic vs. constant model: 15.1, p-value = 6.01e-12

Supplementary Figure 1

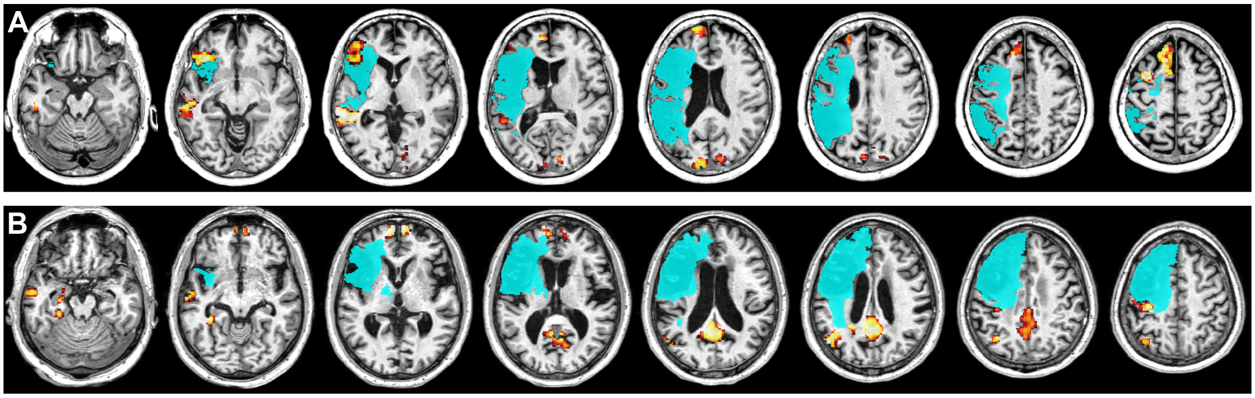

Detailed view of example patient lesions and functional activations. (A) Axial slices showing the lesion and language map for the individual depicted in Figure 4B. (B) Axial slices showing the lesion and language map for the individual depicted in Figure 4C.
